# Macrophage Polarization as a Facile Strategy to Enhance Efficacy of Macrophage Membrane‐Coated Nanoparticles in Osteoarthritis

**DOI:** 10.1002/smsc.202100116

**Published:** 2022-01-29

**Authors:** Kristeen Ye Wen Teo, Cansu Sevencan, Yi Ann Cheow, Shipin Zhang, David Tai Leong, Wei Seong Toh

**Affiliations:** ^1^ Faculty of Dentistry National University Centre for Oral Health National University of Singapore 9 Lower Kent Ridge Road, #10-01 Singapore 119085 Singapore; ^2^ Department of Chemical and Biomolecular Engineering Faculty of Engineering National University of Singapore 4 Engineering Drive 4 Singapore 117585 Singapore; ^3^ Integrative Sciences and Engineering Program NUS Graduate School National University of Singapore 21 Lower Kent Ridge Road Singapore 119077 Singapore; ^4^ Department of Orthopaedic Surgery Yong Loo Lin School of Medicine National University of Singapore NUHS Tower Block Level 11, 1E Kent Ridge Road Singapore 119288 Singapore; ^5^ Department of Biomedical Engineering Faculty of Engineering National University of Singapore 4 Engineering Drive 3 Block 4, #04-08 Singapore 117583 Singapore; ^6^ NUS Tissue Engineering Program Life Sciences Institute National University of Singapore 28 Medical Drive Singapore 117456 Singapore

**Keywords:** cartilage, cell membrane, inflammation, macrophages, nanoparticles, osteoarthritis

## Abstract

Osteoarthritis (OA) is a chronic degenerative joint disorder associated with pain and inflammation, and is the leading cause of disability worldwide. Owing to the complexity of OA inflammation driven by a plethora of inflammatory cytokines, current specific anti‐cytokine therapies have not been successful. Among the immune cells implicated in OA inflammation, macrophages reportedly regulate OA inflammation via macrophage polarization. Given that pro‐inflammatory M1 and anti‐inflammatory M2 macrophages have opposing roles in OA inflammation, exploiting advanced polarization of macrophages to specific macrophage subsets (M0, M1, and M2) to enhance the therapeutic efficacy of macrophage membrane‐coated gold (Au) nanoparticles (NPs) as a broad‐spectrum anti‐inflammatory agent for OA treatment is proposed. Herein, it is shown that among the macrophage membrane‐coated NPs generated from the various macrophage subsets, M2 macrophage membrane‐coated nanoparticles (Au‐M2 NPs) uniquely exhibit superior efficacy in sponging the pro‐inflammatory cytokines and alleviating OA inflammation and matrix degradation over its counterparts derived from the same macrophage cell source, in both inflammation‐stimulated chondrocyte and explant OA models. Collectively, the herein described results validate macrophage polarization as a facile strategy to enhance the therapeutic efficacy of macrophage membrane NP‐based immunotherapy for potential OA treatment.

## Graphical abstract

By coupling cell membrane‐coating nanotechnology with advanced macrophage polarization, it is shown that among various macrophage subsets, M2 macrophage membrane‐coated gold nanoparticles (Au‐M2 NPs) exhibit superior efficacy in capturing pro‐inflammatory cytokines, counteracting inflammation and matrix degradation in chondrocytes and cartilage explants, thus validating macrophage polarization as a facile strategy to enhance the efficacy of macrophage membrane‐coated NPs for potential osteoarthritis (OA) treatment.

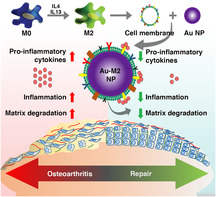

## 1 Introduction

Osteoarthritis (OA) is the most common form of arthritis and one of the leading causes of disability, affecting over 300 million people worldwide.^[^ ^1^ ^]^ With the aging and obesity epidemic, the number of people afflicted with OA is anticipated to increase, along with the increased burden of pain, disability, loss of productivity, and the overall strain of the healthcare system.^[^ ^2^ ^]^ OA is characterized by chronic inflammation and degradation of the joint, including the articular cartilage and subchondral bone.^[2a]^ Although OA frequently occurs at the knee joint, the disease can also affect other joints in the body, including hip joint and temporomandibular joint (TMJ), and is presently one of the major reasons for joint replacement surgeries.^[2a]^

While several risk factors, such as aging, obesity, overuse‐induced joint injury, and genetics, have been linked to OA,[^2^, ^3^] inflammation remains a major cause of OA pathogenesis.^[^ ^4^ ^]^ Despite the evident role of inflammation in OA, recent clinical trials evaluating the use of specific anti‐cytokine therapies to inhibit interleukin (IL)1β or tumor necrosis factor (TNF)α have demonstrated limited efficacy within the OA pathological milieu.^[^ ^5^ ^]^ As OA pathological inflammation is driven by a plethora of inflammatory mediators, including cytokines, matrix metalloproteinases (MMPs), reactive oxygen, and nitrogen species, inhibition of one or a few inflammatory mediators may not be sufficient to halt or reverse the disease progression. The unsuccessful clinical trials hint for any magic bullet approach would require an all‐encompassing broad‐spectrum anti‐inflammatory strategy for OA treatment.

Recently, cell membrane cloaking has emerged as a versatile tool for engineering functionalized nanoparticles (NPs) for therapeutic applications.^[^ ^6^ ^]^ Coating the NPs with cell membrane, endow them with the source cell's rich repertoire of specific receptors and ligands for absorption and neutralization of pathological molecules, sometimes even without specific information of the actual receptors–ligands partners’ identities. This nanomedicine strategy has gained popularity as a powerful therapeutic approach not only in cancer therapy^[^ ^7^ ^]^ but also more recently in the potential treatment of various inflammatory diseases. Various immune cells, such as the neutrophils and macrophages, have been harnessed for the synthesis of membrane‐coated NPs and demonstrated efficacy for neutralization of inflammatory cytokines in the treatment of inflammatory diseases, such as rheumatoid arthritis,^[^ ^8^ ^]^ renal ischemia‐reperfusion injury,^[^ ^9^ ^]^ acute pancreatitis,^[^ ^10^ ^]^ ulcerative colitis,^[^ ^11^ ^]^ atherosclerosis,^[^ ^12^ ^]^ and acute lung injury.^[^ ^13^ ^]^

These prior work inspired us to develop cell membrane‐coated NPs as a broad‐spectrum anti‐inflammatory agent to overcome the complexity of OA inflammation driven by a plethora of inflammatory mediators. Among the immune cells implicated in OA inflammation, macrophages represent one of the prominent innate immune cell types that rapidly infiltrate the inflamed synovium in early OA, mediating joint inflammation and tissue damage.^[^ ^14^ ^]^ Depending on the microenvironmental stimuli, naïve macrophages (M0) can be polarized into two main subsets of macrophages: pro‐inflammatory M1 and anti‐inflammatory M2 macrophages. The M1 macrophages are activated by interferon (IFN)γ or lipopolysaccharide (LPS) to produce other pro‐inflammatory mediators such as IL1β and TNFα that aggravate inflammation and exacerbate tissue damage. In contrast, the M2 macrophages are activated by IL4 or IL13 to produce anti‐inflammatory cytokines such as IL10 and transforming growth factor (TGF)β and contribute vastly to the resolution of inflammation and tissue healing.^[^ ^15^ ^]^ In OA, excessive production of pro‐inflammatory cytokines by M1 macrophages and reduced production of anti‐inflammatory cytokines by M2 macrophages drive the disease pathology and progression.^[^ ^16^ ^]^ Accordingly, a higher ratio of M1 macrophages was reported in the knee of OA patients as compared to healthy control, and was positively associated with the severity of the disease.^[^ ^16^ ^]^ Consistently, M1 macrophages in OA synovium were reported to inhibit chondrogenesis via IL6,^[^ ^17^ ^]^ while M2 macrophages supported the survival of cartilage graft by the production of IL10 to suppress adverse inflammation.^[^ ^18^ ^]^

Given that M1 and M2 macrophages have opposing roles in OA inflammation, we, therefore, postulate that macrophage membrane‐coated NPs generated from the various macrophage subsets could have differential anti‐inflammatory properties that could be harnessed for potential OA treatment. Here, we designed and synthesized macrophage membrane‐coated NPs based on naïve M0, M1, and M2 macrophages’ plasma membranes and investigated their efficacy for broad‐spectrum capture of anti‐inflammatory cytokines, as well as for alleviation of OA inflammation and matrix degradation.

## 2 Results and Discussion

**Figure 1:**
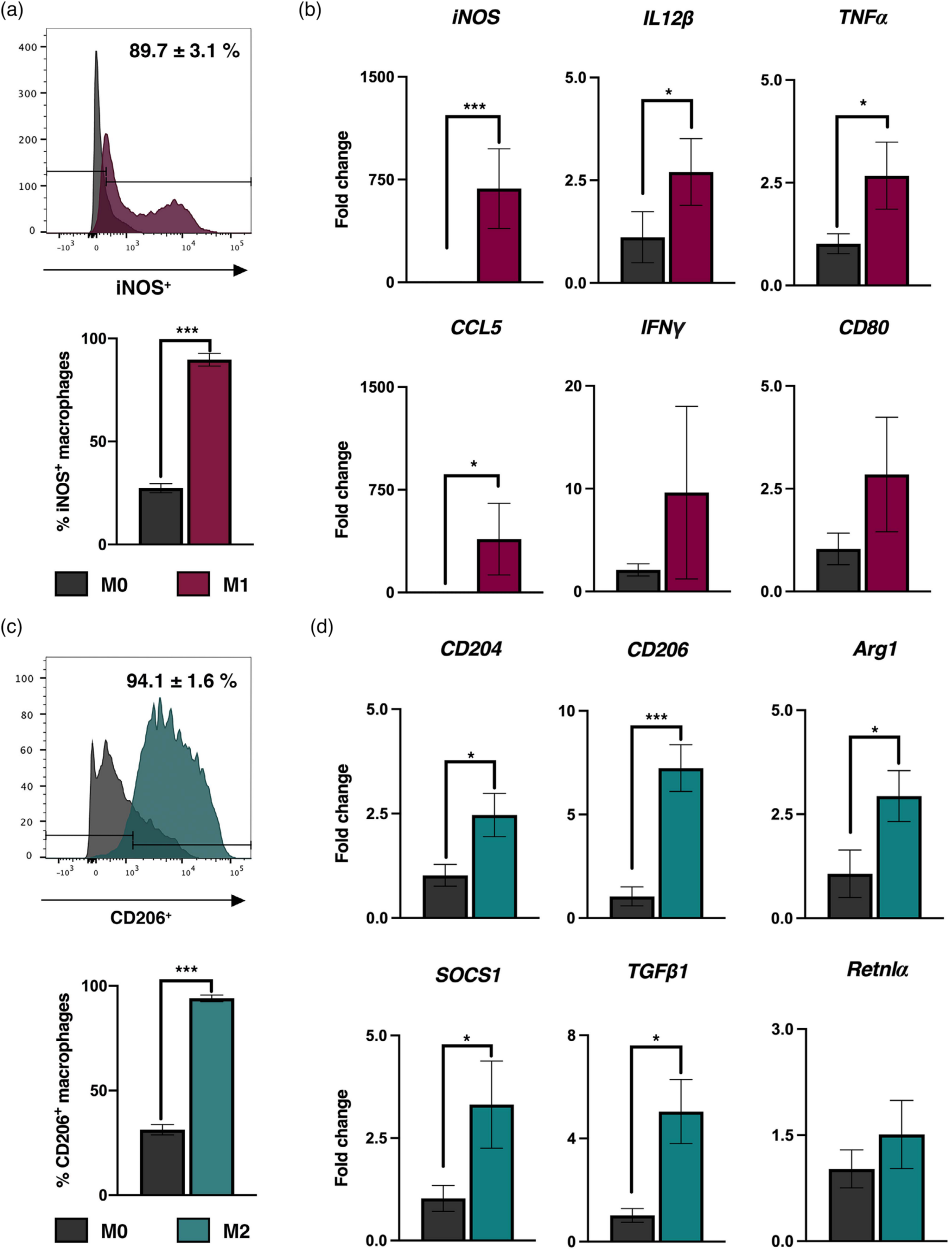
Polarization of naïve M0 macrophages into M1 and M2 subsets. a) Flow cytometry analysis of putative M1 marker iNOS in M0 and M1 polarized macrophages accompanied by b) gene expression analysis of *iNOS*, *IL12β*, *TNFα*, *CCL5*, *IFNγ*, and *CD80*. c) Flow cytometry analysis of M2 marker, CD206 in M0 and M2 polarized macrophages accompanied by d) gene expression analysis of *CD204*, *CD206*, *Arg1*, *SOCS1*, *TGFβ1*, and *Retnlα*. Student's *t*‐test, ^*^ *P* < 0.05, ^**^ *P* < 0.01, ^***^ *P* < 0.001 compared to M0 macrophage group. Data are presented as mean ± SD. *n* = 3/group.

Our findings show that rat macrophages (NR8383) can be effectively polarized under specific culture conditions into pro‐inflammatory M1 or anti‐inflammatory M2 macrophage subsets at high efficiency (**Figure** **1**). Specifically, naïve M0 macrophages treated with IFNγ and LPS were polarized to iNOS^+^ M1 macrophages at high efficiency of 89.7 ± 3.1% (Figure 1a) with significantly increased levels of M1 markers including *iNOS*, *IL12β*, *TNFα*, and *CCL5* (Figure 1b). The other two M1 markers *IFNγ* and *CD80* showed increased levels amid no significant differences. In contrast, naïve M0 macrophages treated with IL4 and IL13 were polarized to CD206^+^ M2 macrophages at high efficiency of 94.1 ± 1.6% (Figure 1c) with significantly increased expression of *CD204*, *CD206*, *Arg1*, *SOCS1*, and *TGFβ1*. No significant difference was noted for *Retnlα* (Figure 1d).

Using an established protocol for NP synthesis,^[^ ^19^ ^]^ we then harvested the membranes from the various macrophage subsets (M0, M1, and M2), and coated them onto the gold (Au) NPs to generate the different macrophage membrane‐coated NPs, Au‐M0, Au‐M1, and Au‐M2, respectively. Au NPs were chosen as a model NP system for this study, due to their good biocompatibility and common use in biomedical applications.^[^ ^20^ ^]^ Plasma membrane from red blood cells (RBC) was used to synthesize Au‐RBC NPs and served as a nonimmune cell membrane control.^[^ ^21^ ^]^ To detect any nonspecific binding, Au NPs with bovine serum albumin (BSA) corona were used as control.

**Figure 2:**
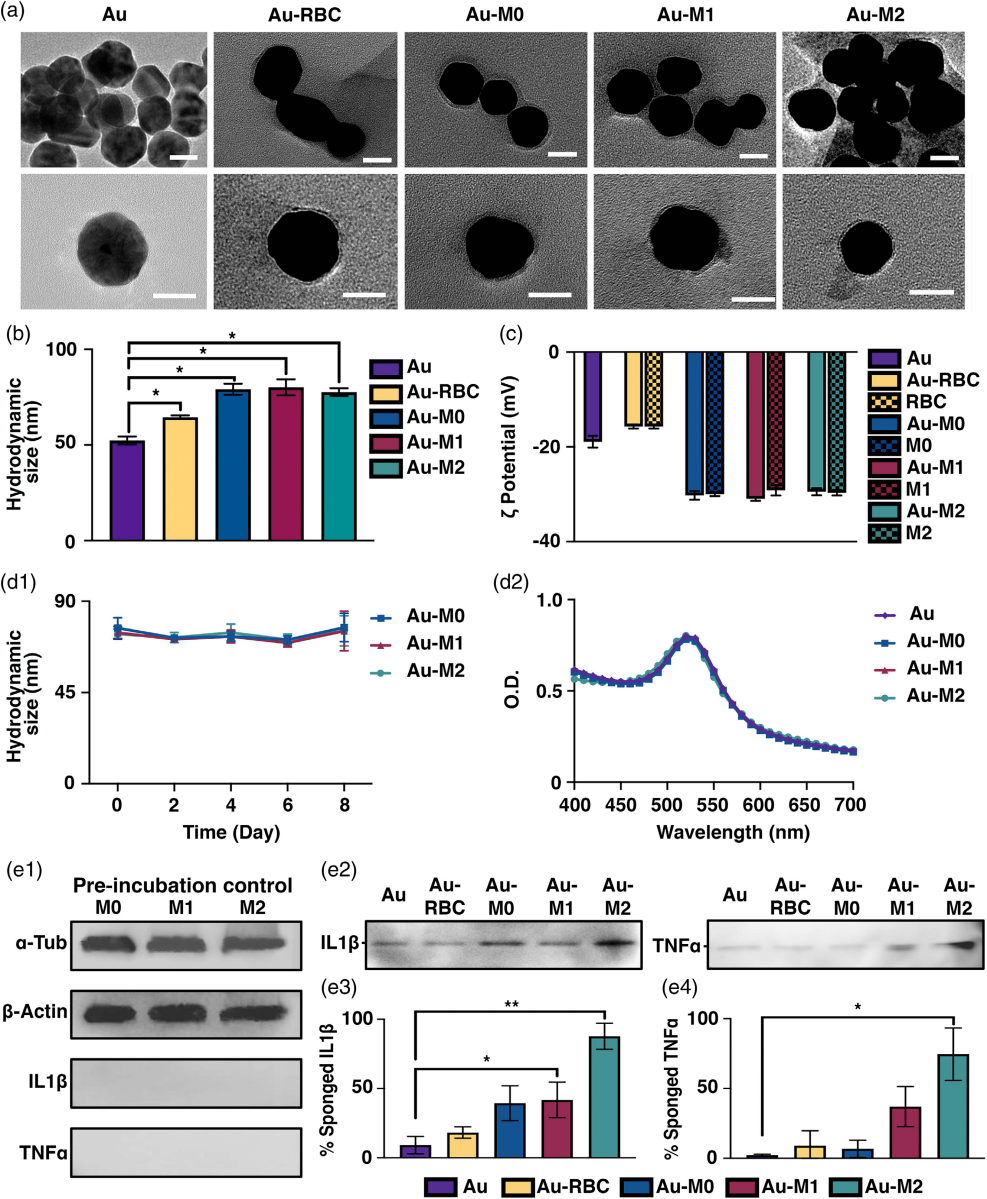
Characterization of cell membrane‐coated NPs. a) TEM images of bare and cell membrane‐coated Au NPs. Scale bar: 20 nm. b) Hydrodynamic size (diameter) and c) zeta potential (ζ) of bare and cell membrane‐coated Au NPs. d1) Stability profile of Au‐M0, Au‐M1, and Au‐M2 NPs in PBS. d2) UV−vis absorption spectra of Au, Au‐M0, Au‐M1, and Au‐M2 NPs. e1) Characteristic protein bands of macrophage membranes resolved by immunoblotting, prior to co‐incubation with IL1β and TNFα. Absence of bands for IL1β and TNFα cytokines shows that the macrophage membranes do not present these cytokines prior to co‐incubation. e2) Immunoblotting analysis of Au, Au‐RBC, Au‐M0, Au‐M1, and Au‐M2 NPs upon 2 h co‐incubation with IL1β or TNFα. Percentage of cytokine sponged following 2 h co‐incubation with e3) IL1β and e4) TNFα. The band intensities were measured and cytokine amounts that corresponded to these band intensities were calculated using standard curves generated from known amounts of cytokines. The values were presented as the percentage of the total amount of cytokines that were used for the co‐incubation experiment. Student's *t*‐test, ^*^ *P* < 0.05, ^**^ *P* < 0.01 compared to Au NPs. Data are presented as mean ± SD. *n* = 3/group.

Transmission electron microscopy (TEM) images showed successful membrane coating of Au NPs in a core–shell structure (**Figure** **2**a). Dynamic light scattering (DLS) measurements also showed the expected hydrodynamic size increase of membrane‐coated NPs in comparison with the bare Au NPs (Figure 2b). Specifically, Au‐M0, Au‐M1, and Au‐M2 NPs increased by ≈25 nm, whereas Au‐RBC NPs increased by ≈10 nm, compared with the bare Au NPs. The polydispersity indexes of the NPs were measured as follows: Au‐M0 (0.301 ± 0.003), Au‐M1 (0.306 ± 0.009), Au‐M2 (0.299 ± 0.003), Au‐RBC (0.307 ± 0.004), and Au NPs (0.465 ± 0.001). The successful membrane coating of the Au NPs was further supported by the surface zeta potential measurement showing that the surface charges of the membrane‐coated Au NPs were comparable to that of individual plasma membranes, suggesting the full surface coverage of the Au NPs with the membranes (Figure 2c). Next, the hydrodynamic size of Au‐M0, Au‐M1, and Au‐M2 NPs in phosphate‐buffered saline (PBS) and without any preservation agents measured by DLS remains at a range of 75–80 nm over 8 days, demonstrating superior colloidal stability of the membrane‐coated Au NPs (Figure 2d1). Additionally, the absorption spectra analysis showed that Au‐M0, Au‐M1, and Au‐M2 NPs have the characteristic absorbance peak of spherical Au NPs, affirming that no significant aggregation occurred during the preparation of the particles (Figure 2d2). Collectively, these results demonstrated the robust physicochemical properties of the macrophage membrane‐coated NPs.

IL1β and TNFα are the predominant pro‐inflammatory cytokines involved in the initiation and progression of articular cartilage destruction in OA.^[^ ^22^ ^]^ Here, we assessed the binding ability of the macrophage membrane‐coated NPs to capture IL1β and TNFα (Figure 2e). Before coating Au NPs with the macrophage membranes, immunoblotting was performed to confirm that these membranes derived from macrophages following polarization do not express any IL1β and/or TNFα (Figure 2e1). Accordingly, the macrophage membranes demonstrated an abundance of housekeeping proteins, including α‐tubulin and β‐actin, but an absence of IL1β and TNFα. Next, we performed co‐incubation of Au‐M0, Au‐M1, Au‐M2, Au‐RBC, and Au NPs with IL1β (200 ng mL^−1^) or TNFα (40 ng mL^−1^) for 2 h. Following incubation, the amounts of bound cytokines to the NPs were assessed by immunoblotting. Among the macrophage membrane‐coated NPs, Au‐M2 NPs showed the strongest band intensities for IL1β and TNFα (Figure 2e2). In contrast, Au‐RBC and Au NPs had very faint bands for both cytokines. Semiquantitative analysis of the protein bands further revealed that Au‐M2 NPs were able to capture 87.7% ± 9.4% and 74.5% ± 18.7% of IL1β and TNFα, respectively, while the capture efficiency through nonspecific binding of Au NPs could only reach 9.17% ± 6.3% for IL1β and 2.29% ± 0.6% for TNFα (Figure 2e3,e4). Additionally, Au‐M2 NPs showed significantly better capture ability compared to Au‐M1 NPs for both IL1β (87.7 ± 9.4% versus 41.8 ± 12.8%, *P* = 0.015) and TNFα (74.5 ± 18.7% versus 37 ± 14.3%, *P* = 0.025). Collectively, these findings demonstrated that among the various macrophage membrane‐coated NPs, Au‐M2 NPs with the membrane derived from M2 macrophages had the most potent cytokine sponging ability, supporting macrophage polarization as a facile strategy to enhance the cytokine sponging abilities of the macrophage membrane‐coated NPs.

**Figure 3:**
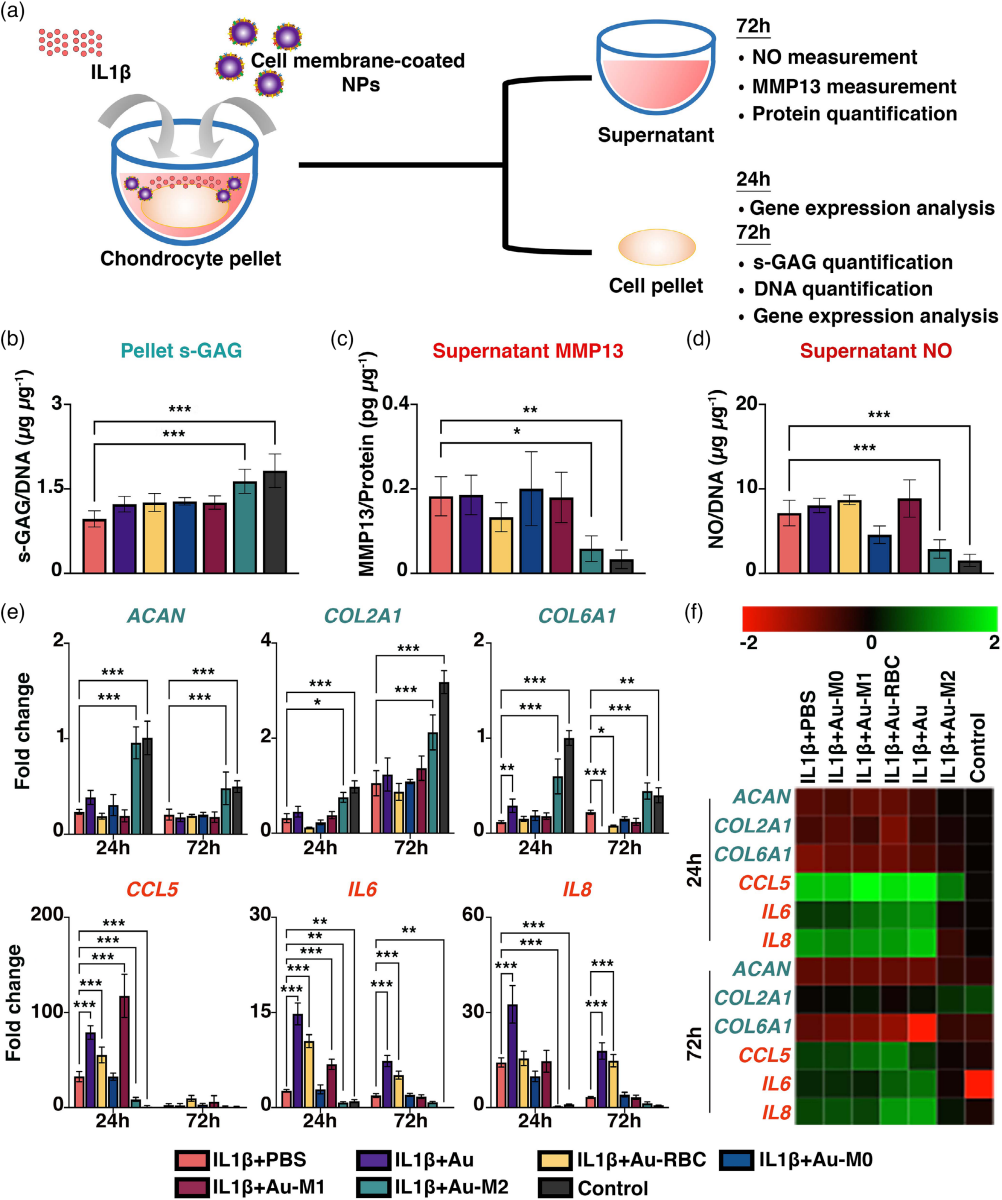
Au‐M2 NPs ameliorate IL1β‐induced inflammation and matrix degradation in chondrocytes. a) Time schedule for the treatment of chondrocyte pellets in vitro. Chondrocyte pellets were treated with or without 1 ng mL^−1^ IL1β along with different cell membrane‐coated NPs and harvested at 24 and 72 h post‐treatment. b) Quantitative analysis of s‐GAG/DNA from chondrocyte pellets at 72 h after treatment. Measurement of c) MMP13 and d) NO from culture supernatants at 72 h after treatment. e) Gene expression analysis of *ACAN*, *COL2A1*, *COL6A1*, *IL6*, *IL8*, and *CCL5* in chondrocyte pellets at 24 and 72 h post‐treatment. f) Heatmap showing nearest neighbor analysis with different treatments using Pearson correlation of gene expression data. One‐way ANOVA, ^*^ *P* < 0.05, ^**^ *P* < 0.01, ^***^ *P* < 0.001 compared to IL1β + PBS group. Data are presented as mean ± SD. *n* = 5/group.

Having affirmed the cytokine sponging ability of the macrophage membrane‐coated NPs, we next examined if these macrophage membrane‐coated NPs could alleviate IL1β‐induced inflammation and matrix degradation of chondrocytes using a chondrocyte OA model previously established.^[^ ^23^ ^]^ As predominant pro‐inflammatory cytokines driving OA progression, IL1β and TNFα mediate matrix degradation through elevated catabolic activities, including nitric oxide (NO) production and MMP activities.^[^ ^22^ ^]^ Here, chondrocytes were seeded as pellets and co‐treated with IL1β and various NP formulations for over 72 h (**Figure** **3**a). In the presence of IL1β, there was reduced sulfated glycosaminoglycan (s‐GAG) synthesis/retention in chondrocyte pellets as evidenced by decreased s‐GAG/DNA in IL1β‐treated chondrocytes but was reversed by Au‐M2 treatment that effectively maintained s‐GAG/DNA at a level comparable to that of untreated control at 72 h (1.64 ± 0.21 versus 1.82 ± 0.30 μg μg^−1^, *P* > 0.05) (Figure 3b). Additionally, Au‐M2 NP treatment effectively abrogated IL1β‐induced MMP13 production. At 72 h, the IL1β‐treated chondrocytes showed a significant ≈70% reduction in MMP13 production following Au‐M2 NP treatment (*P* = 0.01), reaching a level close to the untreated control (0.06 ± 0.03 pg μg^−1^ versus 0.03 ± 0.02 pg μg^−1^, *P* > 0.05) (Figure 3c). Similarly, Au‐M2 NP treatment significantly suppressed NO production that was elevated by IL1β stimulation. Following Au‐M2 NP treatment for 72 h, NO production by IL1β‐treated chondrocytes was significantly reduced by ≈60%, reaching a level comparable to the control (Figure 3d). This functional ability of Au‐M2 to counteract IL1β‐induced inflammation and matrix degradation in chondrocytes appears to be specific to the M2 macrophage membrane as no apparent effects were observed with Au‐M0 and Au‐M1 NP treatments (Figure 3b–d).

The abovementioned findings were further substantiated with our gene expression analysis. Here, we measured the mRNA level of anabolic markers including cartilage matrix genes *ACAN*, *COL2A1*, and *COL6A1*, as well as catabolic markers including pro‐inflammatory genes *CCL5*, *IL6*, and *IL8* following treatment for 24 and 72 h. Consistent with the preserved s‐GAG/DNA by Au‐M2 NP treatment (Figure 3b), we observed a significant increase in *ACAN*, *COL2A1*, and *COL6A1*, attaining levels close to that of the control, as early as 24 h that sustained 72 h (Figure 3e). In parallel with the suppressed NO and MMP13 production (Figure 3c,d), significant suppression of pro‐inflammatory genes such as *CCL5*, *IL6*, and *IL8* was observed in IL1β‐treated chondrocytes with Au‐M2 NP treatment as compared to PBS treatment as early as 24 h (*P* < 0.01) (Figure 3e). Interestingly, Au‐M1, Au‐RBC, and Au NP treatments had minimum effects on the anabolic markers, but upregulated the catabolic markers such as *CCL5* and *IL6*. This is somewhat consistent with reports that elements of Au, BSA, RBC, and M1 macrophages could have some pro‐inflammatory effects.^[^ ^21^, ^24^ ^]^ As revealed by the nearest neighbor analysis of the gene expression results, Au‐M2 NP treatment achieved the highest similarity in the gene expression profile as the control group at both 24 and 72 h. In stark contrast, Au‐M0 and Au‐M1 NP treatments exhibited high similarities in the gene expression profile as the IL1β‐treated chondrocytes with PBS treatment (IL1β + PBS group) at both 24 and 72 h (Figure 3f). At this juncture, it is evident from our findings that macrophage polarization plays a significant role in altering the membranes’ interactions with the cytokines, and the various macrophage membrane‐coated NPs (Au‐M0, Au‐M1, and Au‐M2) displayed very distinct functional capabilities. Notably, Au‐M2 NPs outperformed Au‐M0 and Au‐M1 NPs in not only sponging the pro‐inflammatory cytokines but also alleviating OA inflammation and matrix degradation in our chondrocyte OA model.

**Figure 4:**
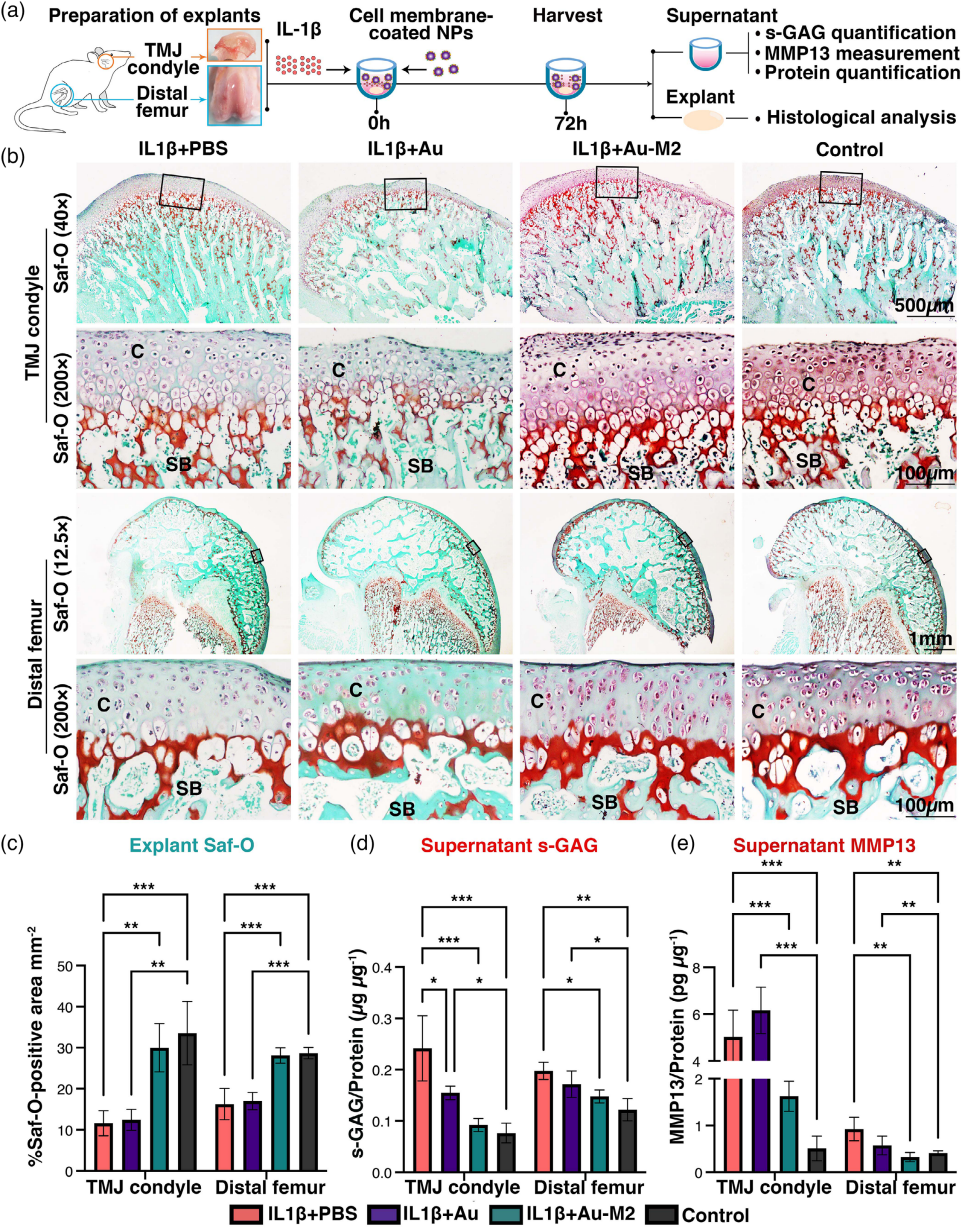
Au‐M2 NPs suppress IL1β‐induced matrix degradation in cartilage explants. a) Time schedule for the treatment of TMJ condyle and distal femur explants. TMJ condyle and distal femur explants were treated with or without 10 ng mL^−1^ IL1β along with Au‐M2 or Au NPs and harvested at 72 h post‐treatment. b) Safranin‐O/fast green (Saf‐O) staining of TMJ condyle and distal femur explants. C, cartilage; SB, subchondral bone. Representative images. Scale bars: 1 mm, 500 μm, or 100 μm. c) Percentage areal deposition of s‐GAG by quantification of Saf‐O‐positively stained area in TMJ condyle and distal femur explants at 72 h post‐treatment. Measurement of d) s‐GAG release and e) MMP13 production in TMJ condyle and distal femur explant culture supernatants at 72 h after treatment. One‐way ANOVA, ^*^ *P* < 0.05, ^**^ *P* < 0.01, ^***^ *P* < 0.001. Data are presented as mean ± SD. *n* = /group.

Finally, to determine the broad applicability of Au‐M2 NPs, we tested their efficacy using rat TMJ condyles and distal femurs in an explant assay (**Figure** **4**a). Here, rat TMJ condyles and distal femurs were co‐treated with IL1β and Au‐M2 NPs for over 72 h. As a result of IL1β‐induced inflammation, there was exacerbated matrix degradation in both TMJ condyles and distal femurs as evidenced by decreased percentage areal deposition of s‐GAG denoted by the Saf‐O‐positively stained area (Figure 4b,c), and increased MMP13 production and s‐GAG loss into the media, when compared to the control groups (Figure 4d,e). These detrimental effects of IL1β‐induced inflammation and matrix degradation were almost reversed with Au‐M2 NP treatment that effectively maintained the percentage areal deposition of s‐GAG in both TMJ condyles and distal femurs at comparable levels as the respective control groups (Figure 4b,c). Consistent with better matrix retention in the explants, Au‐M2 NP treatment significantly reduced the s‐GAG loss by TMJ condyles and distal femurs into the media by ≈60% (*P* < 0.001) and ≈25% (*P* < 0.05), respectively, achieving comparable levels as the control groups. Au‐M2 treatment also concomitantly reduced MMP13 production by TMJ condyles and distal femurs by ≈68% (*P* < 0.001) and ≈65% (*P* < 0.01), respectively, reaching levels comparable to that of the control groups (Figure 4d,e). In contrast, Au NP treatment had no apparent effects on IL1β‐induced matrix degradation and MMP13 production in both TMJ condyles and distal femurs, although a slight inhibitory effect on s‐GAG loss by TMJ condyles was observed (Figure 4b–d). Collectively, these findings demonstrated effective preservation of cartilage matrix with inhibition of s‐GAG loss and MMP13 production with Au‐M2 NP treatment.

Arising from this work, we could attribute the enhanced therapeutic efficacy of Au‐M2 NPs in alleviating OA inflammation and matrix degradation to the improved cytokine sponging capabilities of M2 macrophage membrane over its counterparts derived from the same macrophage cell source. However, there could also be other mechanisms involved that require future investigations. We postulate that the observed effects in our current study could also be due to the differential expression of several surface receptors/proteins caused by macrophage polarization. It has been reported that the expression of membrane proteins CD80 and CD86 upregulated in M1 macrophages was involved in pro‐inflammatory response, as evidenced by a significant reduction in several proinflammatory cytokines including TNF, MIP2, IL1β, IL6, and IL17 in mice lacking CD80 and CD86.^[^ ^25^ ^]^ In contrast, there are several membrane receptors upregulated in M2 polarized macrophages that could exhibit scavenging activities and participate in the neutralization of pro‐inflammatory cytokines. For instance, IL1R2 receptor upregulated in M2 macrophages could act as a decoy in capturing IL1.[^25^, ^26^] Macrophage scavenger receptors such as CD204 (scavenger receptor A), CD206 (mannose receptor), and CD163 are upregulated in M2 macrophages, and these receptors exhibit scavenging activities that facilitate the clearance of harmful toxins/particles.^[^ ^27^ ^]^ These scavenger receptors reportedly enhance phagocytic functions of macrophages to recognize and uptake pathogenic agents, apoptotic host cells, and modified lipoproteins caused by inflammation.^[^ ^27^ ^]^ In addition, surface receptors, anti‐inflammatory cytokines such as IL10^[^ ^28^ ^]^ and TGFβ^[^ ^29^ ^]^ are reportedly present in membrane‐bound form and could contribute to anti‐inflammatory properties of M2 macrophage membranes in alleviating OA inflammation and matrix degradation as observed in our study. Nevertheless, we acknowledge that future studies are needed to decipher the surface antigen differences of macrophage membranes following polarization to inform mechanism‐based translation of macrophage membrane‐coated NPs for OA treatment.

## 3 Conclusion

In this study, we have demonstrated that macrophage membrane‐coated NPs of various macrophage subsets (Au‐M0, Au‐M1, and Au‐M2) generated have differential anti‐inflammatory properties. In particular, M2 macrophage membrane‐coated NPs (Au‐M2) showed superior efficacy in sponging the pro‐inflammatory cytokines and alleviating OA inflammation and matrix degradation over its counterparts derived from the same macrophage cell source, thus validating macrophage polarization as a facile strategy to enhance the therapeutic efficacy of macrophage membrane NP‐based immunotherapy for OA treatment. This enhanced therapeutic efficacy of macrophage‐coated NPs by leveraging on ex vivo advanced macrophage polarization could also potentially be applied in other debilitating inflammatory diseases.

## 4 Experimental Section

4.1

4.1.1

##### Synthesis of Gold NPs

Prior to synthesis, all glassware were cleaned with aqua regia, rinsed with copious ultrapure water and dried. The Au NPs were synthesized following an established stepwise seeded growth method with slight modification.^[^ ^19^ ^]^ Briefly, 1 mL of 25 mм chloroauric acid (HAuCl_4_; Sigma, St. Louis, MO, USA) was added into 150 mL of 2.2 mм sodium citrate solution (C_6_H_5_Na_3_O_7_; Sigma) at 100 °C and mixed by vigorous stirring for 15 min. Afterward, the reaction temperature was brought down to 90 °C, and 1 mL of 25 mм HAuCl_4_ was added twice with 30 min interval. The product of this reaction was taken as generation 0 (G0) and used as a seed for the next step of the stepwise growth of Au NPs. A volume of 55 mL of the G0 was diluted with 53 mL of ultrapure water and mixed with 2 mL of 60 mм C_6_H_5_Na_3_O_7_. This was followed by the addition of 1 mL of 25 mм HAuCl_4_ thrice with 30 min intervals. The product of this reaction was taken as generation 1 (G1) and was used as the seed for the next step following the same method. This process was repeated until generation 4 (G4) is obtained. To remove the excess reactant, the final Au NPs were centrifuged at 12 000 g for 10 min. The gold content of Au NPs was measured by inductively coupled plasma optical emission spectrometry (iCAP 6000 Series, Thermofisher Scientific, Waltham, MA, USA).

##### Macrophage Polarization

NR8383 rat alveolar macrophage cell line (CRL‐2192) was purchased from American Type Culture Collection (ATCC, Manassas, VA, USA). The rat macrophages were cultured and maintained at M0 phenotype in a complete culture medium comprised of Ham's F12K (Kaighn's) medium (Hyclone, Cytiva, MA, USA) supplied with 15% heat‐inactivated fetal bovine serum (FBS; Thermofisher Scientific) and 1% penicillin‐streptomycin (PS; Thermofisher Scientific) at 37 °C with 5% CO_2_. For M1 polarization, the macrophages were treated with 1 ng mL^−1^ rat IFNγ (R&D Systems, Minneapolis, MN, USA) and 25 ng mL^−1^ LPS (InvivoGen, San Diego, California, USA). For M2 polarization, the cells were treated with 10 ng mL^−1^ IL4 (PeproTech, Rocky Hill, NJ, USA) and 10 ng mL^−1^ IL13 (PeproTech). Macrophage polarization was performed in a complete culture medium, and cells without any treatment were considered naïve macrophages (M0) to serve as the control group. After 48 h treatment, the efficiency of macrophage polarization was assessed by flow cytometry and gene expression analysis. All experiments were performed in triplicates (*n* = 3) in at least two independent trials.

##### Flow Cytometry

Flow cytometry was performed to assess the efficiency of macrophage polarization. Forty‐eight hours after polarization, the cells were harvested and resuspended in PBS. To avoid unspecific binding, the cells were preincubated with anti‐CD32 (BD Biosciences, San Jose, CA, USA) for 5 min. Cells were then stained with intracellular DyLight594‐conjugated inducible nitric oxide synthase (iNOS; Novus Biologicals, LLC, USA) and APC‐conjugated anti‐mannose receptor (CD206; Abcam, Cambridge, UK) antibodies using BD Cytofix/Cytoperm kit (BD Biosciences) as per the manufacturer's instruction for 30 min at 4 °C. After staining, the cells were washed three times with Perm/Wash buffer (BD Biosciences) before the fluorescence was quantitated using BD LSRFortessa Cell Analyzer (BD Biosciences). The cells were gated according to their light‐scatter properties to exclude debris and doublets. Results were analyzed using FlowJo (Version 10.4, BD Biosciences).

##### Preparation of Macrophage Membranes

M0, M1, and M2 macrophages were collected by centrifuging at 350 g for 5 min. After washing three times with PBS, they were resuspended in a hypotonic lysis buffer supplied with 1% protease and phosphatase inhibitors (Sigma). The cell suspension was lysed by three consecutive freeze and thaw cycles, followed by 8–10 min sonication using a probe sonicator (Micron TM XL 2000, Qsonica, USA) on ice. The lysed cells were centrifuged at 3200 g for 5 min. The supernatant was collected and further centrifuged at 20 000 g for 25 min to pellet the membrane fragments. Membrane pellets were lyophilized, weighed, and stored at −80 °C for subsequent use. RBC membrane was prepared following an established protocol with slight modification.^[^ ^30^ ^]^ RBCs were pelleted by centrifuging rat blood at 800 g for 5 min at 4 °C. Collected RBCs were washed with ice‐cold PBS three times and lysed in 0.25 × PBS for 20 min in an ice bath. Lysed cells were centrifuged at 1200 g for 5 min at 4 °C and washed twice with ice‐cold PBS.

##### Preparation and Characterization of Membrane‐Coated Gold NPs

Synthesized Au NPs were mixed with macrophage membrane fragments at a ratio of 0.1 mg cell membrane (dry weight) to 10 mg Au NPs. The mixture was extruded through 400 nm polycarbonate membrane using Avanti mini extruder (Avanti Polar Lipids, USA). The membrane‐coated Au NPs were collected by centrifuging at 10 000 g for 10 min. The morphology of bare and membrane‐coated Au NPs was imaged by field emission transmission electron microscopy (JEOL JEM 2010, Japan). The hydrodynamic diameter and zeta potential were measured by DLS (Malvern, UK). The hydrodynamic size of the NPs in PBS was measured by DLS over a period of 8 days to monitor their stability. The absorbance spectra were obtained through spectra scanning from a wavelength of 400 to 700 nm with a microplate reader (BioTek, Winooski, VT, USA).

##### Quantification of Cytokine Sponging

A quantity of 10 mg of Au‐M0, Au‐M1, Au‐M2, Au‐RBC, and Au NPs was incubated with IL1β (200 ng mL^−1^) or TNFα (40 ng mL^−1^) for 2 h. Prior to co‐incubation with either cytokine, Au NPs were pre‐incubated with 5% BSA solution to establish a protein corona mimicking the in vivo condition. A protein corona is known to form on bare Au NPs upon in vivo administration.^[^ ^31^ ^]^ Serum albumin was chosen to mimic this effect as it was reported to be the most abundant protein forming the protein corona.^[^ ^32^ ^]^ Upon 2 h co‐incubation with either cytokine, the NPs were collected by centrifuging at 10 000 g for 10 min. The proteins were extracted with Laemmli's buffer, separated by electrophoresis on 12% sodium dodecyl sulphate (SDS) polyacrylamide gels and subjected to immunoblotting analysis. The band intensities were quantified using ImageJ software (National Institutes of Health, Bethesda, MD, USA). The cytokine amounts that corresponded to these band intensities were calculated using standard curves generated from known amounts of cytokines. Experiments were repeated in three independent trials.

##### Immunoblotting

Proteins were extracted from 0.1 mg of macrophage membranes using Laemmli's buffer, separated by electrophoresis on 12% SDS polyacrylamide gels, electroblotted onto a nitrocellulose membrane, and probed with primary antibodies including anti‐IL1β (Abcam) and anti‐TNFα (Biorad, Hercules, CA, USA) overnight at 4 °C. Thereafter, the samples were washed and incubated with the horseradish peroxidase (HRP)‐conjugated secondary antibody (Santa Cruz Biotechnologies, Dallas, TX, USA) for 1 h. The protein bands were visualized using the Immobilon Western Chemiluminescent HRP substrate (Merck Millipore, Burlington, MA, USA) and then documented using the Syngene Chemiluminescence Imaging System (Synoptics, UK).

##### Chondrocyte OA Model

All procedures were performed according to the Institutional Animal Care and Use Committee at the National University of Singapore under protocol number (R18‐1295). Rat primary condylar chondrocytes were isolated from 8‐week‐old female Sprague‐Dawley rats as previously described.^[^ ^33^ ^]^ Briefly, the dissected condylar cartilage was digested in 0.2% (w/v) collagenase I and II (Worthington, Lakewood, NJ, USA) for 4 h at 37 °C. Single cells were obtained through a 40 μm nylon cell strainer (Corning, NY, USA) and cultured at a density of 2 × 10^4^ cells cm^−2^ in dulbecco's modified eagle medium (DMEM)/F12 (Hyclone) supplemented with 10% FBS, 1% PS, and 25 μg mL^−1^ L‐ascorbic acid‐2‐phosphate (AA2P; Sigma) at 37 °C with 5% CO_2_. Upon confluence, chondrocytes were dissociated using TrypLE (Thermofisher Scientific) and further sub‐cultured to passage (P) 2 cells for experiments. To assess the chondroprotective effects of different membrane‐coated NPs, an in vitro chondrocyte OA model utilizing P2 chondrocytes stimulated with IL1β was employed as previously described.^[^ ^23^ ^]^ Briefly, 2.5 × 10^5^ cells were seeded each well in 96‐well U‐bottom plates and centrifuged at 300 g for 5 min. The cells were cultured in the serum‐free chondrogenic medium^[^ ^34^ ^]^ composed of high glucose DMEM (Hyclone) supplemented with 1% insulin‐transferrin‐selenium (ITS + 1; Sigma), 1 mм sodium pyruvate (Thermofisher Scientific), 2 mм GlutaMAX (Thermofisher Scientific), 40 μg mL^−1^ L‐proline (Sigma), 50 μg mL^−1^ AA2P, 10^−7^ m dexamethasone (Sigma), 10 ng mL^−1^ TGF‐β1 (R&D Systems), and 1% PS at 37 °C with 5% CO_2_. The resulting chondrocyte pellets were stimulated with 1 ng mL^−1^ IL1β (PeproTech) and co‐treated with different cell membrane‐coated NPs (50 μg mL^−1^) or vehicles (PBS) for over 72 h. Pellets without IL1β stimulation served as control. The pellets were harvested at 24 and 72 h post‐treatment for subsequent analyses. Cell culture supernatant was also collected and centrifuged at >10 000 g for 10 min to remove residual NPs. Experiments were performed using five pellets/group in two independent trials.

##### Explant OA Model

Sixteen TMJ condyles with a mean weight of 9.32 ± 2.28 mg and sixteen distal femurs with a mean weight of 360.79 ± 31.29 mg were dissected from eight 8‐week‐old female Sprague‐Dawley rats after euthanasia and washed with PBS. The TMJ condyle and distal femur explants were then cultured in serum‐free DMEM/F12 with 1% PS and co‐treated with 10 ng mL^−1^ rat IL1β (PeproTech) and Au‐M2 NPs (10 μg mL^−1^) or equivalent amount of Au NPs or PBS for 72 h. Explants cultured in serum‐free DMEM/F12 with 1% PS were used as control. By the end of 72 h, the explants were harvested for histological and histomorphometric analyses. The culture supernatant was collected for s‐GAG, MMP13, and protein quantification. All explant experiments were performed in quadruplicate (*n* = 4).

##### Quantitative Real‐Time Reverse Transcription Polymerase Chain Reaction (qRT‐PCR)

Total RNA was isolated using PureLink RNA Mini kit (Thermofisher Scientific) according to the manufacturer's instruction. The RNA was reverse transcribed to cDNA using iScript reverse transcriptase Supermix (Bio‐Rad Laboratories, Hercules, CA, USA) and then amplified using CFX Connect real‐time PCR system with iTaq Universal SYBR Green Supermix (Bio‐Rad). The PCR cycling condition comprised an initial denaturation at 95 °C for 30 s followed by 40 cycles of amplification consisting of a 15 s denaturation at 95 °C for 30 s and a 30 s of extension at 60 °C. The primers are listed in **Table** **1**. Relative mRNA expression levels were normalized against glyceraldehyde 3‐phosphate dehydrogenase (GAPDH), calculated using the comparative ^Δ^CT method, and finally expressed as fold changes. Heatmap for transcriptional profiling of chondrocyte pellets was generated using the Morpheus online software (https://software.broadinstitute.org/morpheus/) and results were analyzed using the nearest neighbor analysis with different treatments via Pearson correlation of gene expression data.

**Table 1:** Primer sequences

Gene	Primer	Sequence (5’ to 3’)
*ACAN*	Forward	CAGAACCTTCGCTCCAATGAC
	Reverse	CCTCAATGCCATGCATCACTT
*Arg1*	Forward	TTGATGTTGATGGACTGGAC
	Reverse	TCTCTGGCTTATGATTACCTTC
*CCL5*	Forward	CGTGAAGGAGTATTTTTACACCAGC
	Reverse	CTTGAACCCACTTCTTCTCTGGG
*CD204*	Forward	ACAAGGTACTGGTCCAATATG
	Reverse	TGTGAACAGCCTAATTCTCC
*CD206*	Forward	CTCTAAGCGCCATCTCCGTT
	Reverse	CATGATCTGCGACTCCGACA
*CD80*	Forward	CCAAGTGTCCAAGTCGGTGA
	Reverse	TTGTACTCGGGCCACACTTC
*COL2A1*	Forward	CCCCTGCAGTACATGCGG
	Reverse	CTCGACGTCATGCTGTCTCAAG
*COL6A1*	Forward	CCCTGGTGGACAAGGTGAAA
	Reverse	CGCATGAGCCCTCTGATGAT
*GAPDH*	Forward	GGTCGGTGTGAACGGATTTGG
	Reverse	GCCGTGGGTAGAGTCATACTGGAAC
*IFNγ*	Forward	GATCCAGCACAAAGCTGTCA
	Reverse	GACTCCTTTTCCGCTTCCTT
*IL6*	Forward	TTGCCTTCTTGGGACTGATG
	Reverse	GCCATTGCACAACTCTTTTC
*IL8*	Forward	CATTAATATTTAACGATGTGGATGCGTTTCA
	Reverse	GCCTACCATCTTTAAACTGCACAAT
*IL12β*	Forward	CCGATGCCCCTGGAGAAAC
	Reverse	CCTTCTTGTGGAGCAGCAG
*iNOS*	Forward	GAAACTTCTCAGCCACCTTGG
	Reverse	CCGTGGGGCTTGTAGTTGAC
*Retnlα*	Forward	CAACAGGATGAAGACTGCAACCT
	Reverse	GGGACCATCAGCTAAAGAAG
*SOCS1*	Forward	GCAGCTCGAAGAGGCAGTCGA
	Reverse	GCTCCCACTCTGATTACCGGCG
*TGFβ1*	Forward	AAGAAGTCACCCGCGTGCTA
	Reverse	TGTGTGATGTCTTTGGTTTTGTCA
*TNFα*	Forward	CCAGGTTCTCTTCAAGGGACAA
	Reverse	GGTATGAAATGGCAAATCGGCT

##### Quantification of s‐GAG, NO, DNA, and Protein

For s‐GAG measurement, chondrocyte pellets were digested with proteinase K digestion buffer (100 μg mL^−1^ in Tris‐HCl buffer, pH 8) for 18 h at 60 °C before analyzed using Biocolor Blyscan Glycosaminoglycan Assay Kit (Biocolor Ltd, Carrickfergus, UK), following the manufacturer's instruction. For s‐GAG released by cartilage explants, culture supernatants were collected for s‐GAG measurement. The absorbance in standards and samples were measured at 656 nm using a microplate reader (Spark, Tecan, Männedorf, Switzerland) and the amount of s‐GAG was calculated. The accumulation of NO in the cell culture supernatant was measured using Griess Reagent Kit (Thermofisher Scientific) according to the manufacturer's instruction. Briefly, 150 μL of standard or supernatant samples were incubated with 20 μL of Griess reagent and 130 μL of deionized water for 30 min at room temperature. The absorbance at 548 nm was measured and the nitrite concentration was calculated using the standard curve. DNA concentration that is reflective of the cell number was measured using Quant‐iT Picogreen dsDNA assay kit (Thermofisher Scientific) with fluorescence readings taken at excitation 480 nm and emission 520 nm. Total protein concentration from culture supernatant was measured using Pierce Coomassie Protein Assay Kit (Thermofisher Scientific) according to the manufacturer's instruction and readings were taken at 595 nm. The s‐GAG and NO concentrations of chondrocyte pellets were both normalized against the DNA concentration and presented as s‐GAG/DNA and NO/DNA, respectively. The released s‐GAG that is reflective of matrix degradation by the cartilage explants was normalized against total protein and presented as s‐GAG/protein.

##### MMP13 Assay

MMP13 present in culture supernatants of chondrocyte pellets and cartilage explants at 72 h post‐treatment was measured using a rat MMP13 enzyme‐linked immunosorbent assay kit (MyBiosource, San Diego, CA, USA) following the manufacturer's instructions. Absorbance readings at 450 nm were obtained using a microplate reader (Spark, Tecan). The MMP13 concentration in the culture supernatant was normalized against total protein and presented as MMP13/protein.

##### Histological and Histomorphometric Analyses

The harvested explants were first fixed in 10% (v/v) neutral buffered formalin for 3 days followed by decalcification in 30% (v/v) formic acid for 7 days. The specimens were then dehydrated and embedded in paraffin for microtomy following standard procedure.^[^ ^6^ ^]^ Serial 5 μm sections were cut and stained with safranin‐O/fast green (Saf‐O) for s‐GAG deposition as previously described.^[^ ^7^ ^]^ The stained sections were then imaged using an inverted microscope (Olympus IX70; Olympus, Tokyo, Japan). The percentage of Saf‐O‐positively stained area for s‐GAG in the cartilage layer was measured using ImageJ software and expressed as % Saf‐O‐positive area mm^−2^.

##### Statistical Analysis

The distribution of datasets was checked using tests of normality. All data were expressed as mean ± standard deviation (SD). Differences between groups were determined by Kruskal–Wallis test followed by Bonferroni post hoc test for non‐normally distributed data, and by one‐way analysis of variance (ANOVA) followed by Bonferroni post hoc test for normally distributed data. SPSS version 26.0 (SPSS, Chicago, IL, USA) was used to perform statistical analysis. Statistical significance was set as *P* < 0.05. Nearest neighbor analysis via Pearson correlation was performed using Morpheus online software (https://software.broadinstitute.org/morpheus/).

## Conflict of Interest

The authors declare no conflict of interest.

## Data Availability

The data that support the findings of this study are available from the corresponding author upon reasonable request.
